# Prevalence of Metabolic Syndrome Based on the Dietary Habits and Physical Activity of Korean Women Cancer Survivors

**DOI:** 10.3390/foods12193554

**Published:** 2023-09-25

**Authors:** Peng Zhou, Yonghwan Kim, Jiseol Lee

**Affiliations:** 1 Department of Physical Education, General Graduate School, Yongin University, Yongin 17092, Republic of Korea; 202171407@yiu.ac.kr; 2 Department of Physical Education, Gangneung-Wonju National University, Gangneung 25457, Republic of Korea

**Keywords:** cancer survivors, dietary habit, metabolic syndrome, physical activity, prevalence

## Abstract

Cancer is a major cause of death in Korea. Improving dietary habits and encouraging physical activity (PA) are important in managing the quality of life and health of patients. Cancer survivors (CS) often exhibit a higher incidence of metabolic syndrome (MetS) than non-cancer (NC) individuals. The purpose of this study was to analyze the prevalence of MetS according to dietary habits and PA in women who survived various cancers: stomach, colorectal, breast, cervical, lung, thyroid, and others. The participants (*n* = 12,676; NC: 11,673, CS: 1003) were analyzed cross-sectionally over a 6-year period. Caloric intake, eating-out frequency, breakfast frequency, dietary supplements, dietary therapy, nutritional education, participation in aerobic activity, strength training frequency, and sedentary lifestyle were evaluated. The prevalence of MetS was 1.22 (95% confidence interval (CI), 1.07–1.39) times higher in CS than in NC, exhibiting a 1.77-fold (95%CI, 1.14–2.74) increase in colorectal cancer, 1.72-fold (95%CI, 1.29–2.30) in cervical cancer, and 3.07-fold (95%CI, 1.14–5.31) in lung cancer. A higher-than-recommended caloric intake and frequent eating out increased MetS 1.43-fold (95%CI, 1.09–1.79) and 1.11-fold (95%CI, 1.01–1.64), respectively, in NC, and 1.31-fold (95%CI, 1.03–1.75) and 2.65-fold (95%CI, 2.29–3.07), respectively, in CS. Aerobic activity below the recommended level resulted in a 1.37-fold (95%CI, 1.13–1.71) and 1.36-fold (95%CI, 1.10–1.87) increase in NC and CS, respectively, whereas muscle strength increased 1.36-fold (95%CI, 1.08–1.70) and 1.49-fold (95%CI, 1.07–2.57), respectively, at below recommended levels. MetS was more prevalent in CS than in NC; high caloric intake, frequent eating out, low PA, and more sedentary time increased the risk of MetS.

## 1. Introduction

Cancer is one of the most common diseases and a leading cause of death in Korea. In 2022, there were 274,488 new cancer cases and 81,277 cancer-related deaths [1]. The age-standardized incidence rate is 253 per 100,000 persons, and ranks among the highest worldwide [2]. Korea is actively developing early cancer screening systems and medical technology, and promoting preventive measures. Nevertheless, the number of patients with cancer has been continuously increasing over the past 20 years, and this trend is expected to continue, owing to the aging of the population [3].

The most common feature of cancer is its high mortality rate. In addition, after treatment, many cancer survivors experience difficulty in resuming normal daily life, and because chronic long-term physical and mental issues may occur, medical expenses and productivity losses are inevitably large [4,5]. The side effects in patients with cancer include functional weakness due to organ resection, side effects associated with long-term drug use, aftereffects of chemotherapy and radiation therapy, and metabolic and hormonal changes [6,7].

MetS is a representative disease that is often reported in cancer survivors (CS). According to a meta-analysis of 9 studies, CS patients had a 1.84-fold increase in MetS compared to non-cancer people. Moreover, MetS increased by 1.94 times for hematologic malignancie CS and 4.29 times for sarcoma [8]. Metabolic changes possibly cause MetS in patients with CS [9]. In addition, there is psychological degradation due to the after effects of treatment, pain, and physical discomfort. This leads to low physical activity (PA) and causes obesity, high blood pressure, and dyslipidemia, which are risk factors for cardiovascular disease. Conversely, the risk of cardiovascular disease improved in patients with CS with high PA levels [10].

A 12-week circuit resistance training program conducted in breast CS resulted in significant improvements in aortic pulse wave velocity compared to the control group [11]. In another study, 36 weeks of combined aerobic and strength training significantly improved low density lipoprotein cholesterol and reduced body fat in the CS group compared with the control group [12]. In addition, increased PA and healthy diets improve eating habits and reduce visceral fat [13]. However, contradictory results have been reported. In a Korean study, the MetS odds ratio (OR) in CS was not significantly different from that in non-cancer (NC) individuals, and stomach CS exhibited a 58% lower MetS prevalence [14]. A health management program conducted in CS for one year improved the quality of life, but there were limitations regarding PA and the management of eating habits [15].

Even so, the participation rate of CS in healthcare programs in Korea is very low, and compared to the high incidence, research on their health behaviors and MetS is limited. Therefore, this topic remains controversial and requires further research. We aimed to analyze the MetS status and factors associated with dietary habits and types of PA in women patients with cancer. Furthermore, this study aims to determine the prevalence of MetS in breast and cervical cancers occurring in women, as well as relatively common stomach, colorectal, lung, and thyroid CS. Through this research, we hope to contribute to improved healthcare provision for CS.

## 2. Materials and Methods

### 2.1. Participants

In the Korean National Health and Nutrition Examination Survey, a government agency conducts an annual survey of voluntary participants of all age groups across the country. The data of the survey participants, who provided consent for clinical tests, data analysis, and publication for research purposes, are disclosed and disseminated through a web page. 

In the questionnaire, the socioeconomic status (income and education level), including diet, PA, smoking, and drinking status, were investigated. The staff provided assistance only if the participants had difficulty understanding the questions or had poor eyesight. As a preliminary preparation for the test, participants fasted for 8 h, and light gowns and slippers were provided for the test.

**Figure 1:**
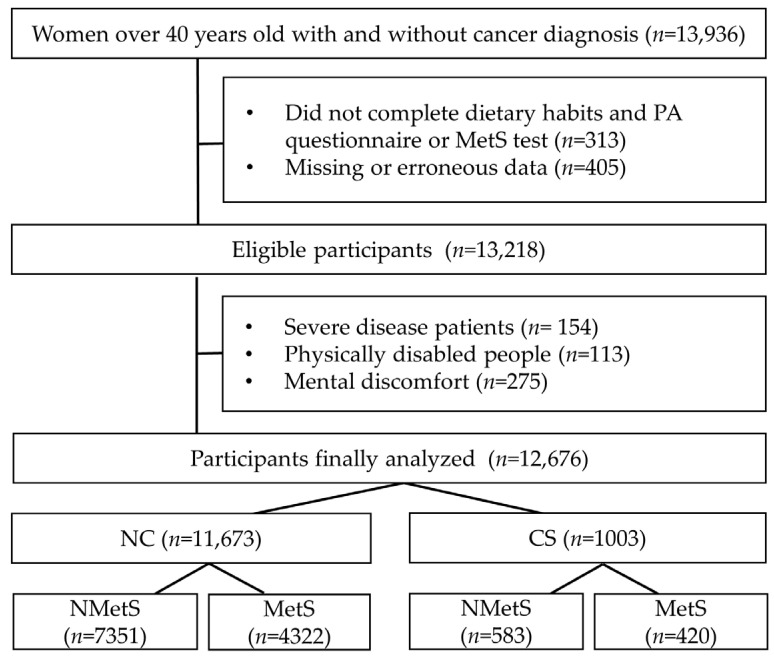
Participant inclusion diagram. NC, non-cancer; CS, cancer survivors; NMetS, non-metabolic syndrome; MetS, metabolic syndrome; PA, physical activity.

Physical and blood pressure (BP) measurements and blood sampling were performed during the clinical examination (Figure 1). This study complied with the guidelines of the Declaration of Helsinki and was approved and supervised by the Research Ethics Committee of Gangneung–Wonju National University (Approval no.: 2020-16).

### 2.2. Health Questionnaire

Regarding socioeconomic status, the education level (middle school, high school, and college) and monthly household income (high, medium, and low) were investigated, and the type, frequency, and amount of drinking were investigated to determine the degree of risk (low, medium, and high) as classified by the World Health Organization (WHO) [16]. Smoking status (current, stopped, or never) was investigated, and the status and years were recorded.

### 2.3. Dietary Habits

We used a dietary habit questionnaire that was developed by the Korea Centers for Disease Control and Prevention and verified for reliability and validity in this study [17]. Only the items relevant to the study were analyzed; the calories consumed per day, number of times eating out per week, frequency of breakfast consumption, and frequency of three meals per day were assessed. In addition, whether participants obtained nutritional education and dietary supplement intake were included. The 24 h recall method was used for calorie intake, and a questionnaire developed by the host institution was used as the food frequency questionnaire [18]. The food frequency questionnaire comprised 112 items. The survey included the consumption frequency or quantity and aligned the data with age-specific daily nutritional recommendations [19]. The nutritional components and calories included in the food composition table were calculated. Participants were also asked whether they had undergone diet therapy for weight loss or illness.

The frequency of three meals per day per week was classified as high (5–7 days/week) or low (0–4 days/week). The weekly frequency of eating out was classified as low (≤4 times/week), medium (5–6 times/week), and high (1–2 times/day). The weekly breakfast frequency was classified as high (5–7 days/week), medium (3–4 days/week), or low (0–2 days/week) [20,21].

### 2.4. Physical Activity

The PA questionnaire was based on the International Physical Activity Questionnaire developed and disseminated by the WHO. We assessed the frequency of strength training [22,23]. The PA questionnaire used a 7-day memory recall method, and the participants completed the questionnaire independently.

The PA frequency and times per week were recorded. The total sedentary time per day was also determined. Sedentary time refers to the time spent sitting and inactive, excluding sleeping. This included the time spent at work, watching TV, and using computers. The variables were classified as low, medium, or high. The level of aerobic satisfaction during PA followed the guidelines of the American College of Sports Medicine, and the recommended level was 3–5 days per week for aerobic and 2–3 days for strength training PA [24].

### 2.5. Diagnosis of Metabolic Syndrome

MetS was diagnosed based on The National Cholesterol Education Program adult criteria. The Treatment Panel III (NCEP ATP III) was applied [25]. Abdominal obesity was measured using the Asian criteria presented by the WHO [26]. The criteria are waist circumference (WC, ≥85 cm), systolic blood pressure (SBP, 130 mmHg) or diastolic blood pressure (DBP, 85 mmHg), glucose (GLU, ≥100 mg/dL), triglyceride (TG, ≥110 mg/dL), high density lipoprotein cholesterol (HDLC, <50 mg/dL), and MetS was diagnosed if the score was ≥3.

Waist circumference was measured horizontally at the thickest part, near the navel, using a tape measure. If an error ≥ 0.5 cm occurred after two measurements, the measurement was repeated and the highest value was used [27]. A nurse collected blood samples from the median cubital vein. A compression band was tied to the upper arm to expose the vein, which was subsequently lightly tapped with a finger. A needle was inserted into the vein and 25–30 mL of blood was collected using a vacuum tube. Blood pressure was sufficiently stable for more than 5 min and was measured three times using an electronic blood pressure monitor. The average value of the second and third measurements was recorded.

### 2.6. Data Analysis

Participants were divided into CS and NC groups. The MetS and non-MetS (NMetS) groups were compared. Data analysis was performed using the SPSS software (version 25.0; SPSS Inc., Chicago, IL, USA). Continuous variables are expressed as means and standard deviations, and categorical variables are expressed as numbers and percentages. An independent *t*-test was used for between-group comparisons, and the chi-square test was used for categorical variables. MetS prevalence was calculated as odds ratios (ORs) using logistic regression analysis. The adjusted variables in the NC group included age, household income, smoking status, and alcohol consumption, and included the participants’ age, household income, and alcohol consumption in the CS group. The significance level was set at *p* < 0.05, and the confidence interval (CI) was 95%.

## 3. Results

### 3.1. General Characteristics

Factors such as age, height, and weight in both the CS and NC groups were significantly different between the MetS and NMetS groups. There were no significant differences in the incidence of MetS according to the number of cancers or duration of cancer survival. Household income and alcohol risk differed significantly according to the presence or absence of MetS in both the NC and CS groups. There was no significant difference in education level in any group, and smoking status was only significantly different in the NC group (Table 1).

### 3.2. Odds Ratio for MetS and Factors According to Cancer Type

The MetS OR of CS was 1.22 times higher than that of the NC group. In particular, there were significant increases in the HDL, TG, and GLU levels. There were no significant differences in ORs in patients with stomach, breast, or thyroid cancers. The MetS OR increased 1.77-fold in colorectal cancer and 1.72-fold in cervical cancer, 3.07-fold in lung cancer, and 1.45-fold in other cancers (Table 2).

### 3.3. MetS Odds Ratio According to Dietary Habits

Commonly, in NC and CS, the OR for MetS increased according to calorie intake, the frequency of three meals per day, frequency of eating out, and nutrition education. A higher-than-recommended caloric intake increased the OR by 1.43 and 1.31 times, respectively, and eating out frequently increased the OR by 1.11 and 2.65 times, respectively.

In the CS group, breakfast frequency and diet therapy were significantly different. The OR for MetS increased 3.37 times in the group with low breakfast frequency, and 1.24 times in the group without diet therapy (Table 3).

### 3.4. MetS Odds Ratio According to Physical Activity

For aerobic PA, OR increased by 1.37 and 1.36 times in the NC and CS groups with levels below the recommended level, respectively, and muscle strength increased by 1.36 and 1.49 times, respectively, at levels below the standard level. However, CS performing aerobic PA above the recommended level lowered the risk of MetS by 12% and, in the group performing strength training PA above the recommended level, the risk was lowered by 22%.

The NC group, with a highly sedentary lifestyle, exhibited a 1.90-fold increase in the OR and the CS group showed a 1.85-fold increase (Table 4).

## 4. Discussion

In CS, health ultimately refers to an individual’s well-being and mental and physical health after cancer treatment. This study aimed to investigate the prevalence of MetS in CS based on the dietary habits and physical activity levels of Korean women.

A representative result of this study was a 1.22-fold increase in the OR for MetS in CS. Cervical, lung, and other cancers showed a significant increase in the incidence of MetS, whereas stomach, breast, and thyroid cancers did not. Studies of the prevalence of MetS in CS have reported conflicting results. In a meta-analysis study that investigated CS in 1762 cases, the risk of MetS increased by 1.84-fold. In addition, MetS increased 2.4 times in acute lymphoblastic leukemia, acute myelogenous leukemia, and chronic myelogenous leukemia CS compared to NC [8]. 

However, the presence of MetS was not significantly associated with CS. In a 2013 study conducted in Korea, no significant increase in the prevalence of MetS was observed in patients with CS; however, the risk of MetS in stomach cancer patients was 58% lower than that in the cancer-free group [14].

Several studies have been conducted showing that metabolic syndrome affects colorectal cancer [28,29], however, studies showing that colorectal CS causes MetS are very rare [14,30]. A study conducted in Korea found that the prevalence of MetS in people with colorectal cancer increased 1.6-fold, a result similar to that of this study [14]. Meanwhile, MetS was reported to increase 2.11-fold and 1.68-fold in lung and thyroid CS, respectively [30].

The mechanisms linking a high risk of MetS to CS have not been fully elucidated. In general, MetS is exacerbated by reduced PA, high-calorie food intake, high fat intake, and low fiber intake. However, these factors are also potential cancer-related markers. In addition, excessive obesity, especially visceral obesity, complicates cancer research, and chronic inflammatory conditions are likely to occur simultaneously in patients with cancer and cardiovascular disease [9,31].

In the CS in this study, the risk of MetS increased 1.6–3.3 times in the group with low breakfast frequency. These results were often reported in previous studies, and their mechanisms have been explained in various ways. Skipping breakfast can lead to a drop in blood sugar levels due to longer periods without food which, over time, can lead to insulin resistance, making cells less responsive to insulin, leading to higher blood sugar levels. Additionally, persons who skip breakfast are more likely to consume high-calorie foods in the afternoon, which may lead to obesity [32,33].

Furthermore, breakfast intake may affect hormones such as ghrelin and leptin, which are involved in metabolism and appetite regulation [34]. Therefore, eating a nutritious breakfast can regulate these hormones and contribute to better appetite control throughout the day.

However, despite various possible explanatory mechanisms, there are still reports that intermittent fasting is beneficial for weight control, and there are also claims that, if the overall calories can be lowered by skipping breakfast, it is beneficial for obesity and MetS [35,36]. Another study showed that skipping breakfast did not affect insulin sensitivity or ghrelin and leptin levels [37].

The relationship between meal frequency and metabolic syndrome factors showed similar results in other studies. Compared to people who consumed one to two meals a day, people who consumed more meals had a lower total cholesterol and low density lipoprotein cholesterol concentration of about 0.25 mmol/L. Additionally, they had healthier eating habits in terms of total energy intake and macronutrient distribution [38]. Additionally, the fasting blood sugar level was higher in the group that ate one meal a day compared to the group that ate three meals, and no benefit was observed in lipid, fasting glucose, and insulin levels in the group that ate one meal a day for 8 weeks compared to those who ate three meals [39]. So, it was reported that people with irregular dietary habits had a 1.7-fold increased risk of cardiovascular disease in a long-term follow-up study [40].

Although the composition of dietary supplements was not investigated in this study, the results showed that dietary supplement intake did not prevent MetS. Previous studies have shown varying results for supplements. Low MetS has been reported in olive oil or oleic acid supplement users [41], and wild bitter gourds reduced MetS [42]. However, the intake of supplements such as fiber did not result in significant changes [43].

The recommended level of PA is 3–5 days of aerobic and 2–3 days of strength training [24]. The NC and CS groups that performed more PA than this recommended value exhibited a lower MetS risk, whereas the group with lower PA had an increased risk of MetS. These results are similar to those of other studies in NC individuals [44,45,46].

The importance of PA is emphasized in patients with cancer. A study in patients with breast cancer showed that PA improved insulin-like growth factor I levels, and strength training was associated with improvements in fatigue, depression, and quality of life. When studies on different types of cancer were analyzed, there were significant improvements in body mass index, body weight, maximal oxygen consumption, maximal strength, 6 min walk time, and quality of life [47].

Moreover, PA in CS has been reported to reduce cancer recurrence and mortality rates. A meta-analysis of 136 studies found that CS with high PA lowered cancer mortality by 18% and CS with breast and colorectal cancers saw a reduction by 37–42% [48].

Ultimately, based on these studies, healthcare providers need to more actively intervene and educate CS about the importance of PA and dietary patterns, and explain the potential long-term side effects and recommended follow-up healthcare. In addition, it is desirable to address the needs of CS and provide guidance for ongoing healthcare. As cancer survival rates continue to improve with the development of medical technology, more focus is being placed on improving the quality of life of CS, which should include addressing physical health issues, managing mental health, promoting healthy lifestyles, and encouraging survival-oriented research. Social support also plays an important role in coping with the physical and emotional challenges faced by CS. Support from family, friends, support groups, and mental health professionals can significantly improve the well-being of patients and enhance coping mechanisms.

Cancer and MetS have been consistently researched; however, there are various limitations. This is because it is not possible to accurately determine the cardiovascular health status of patients before the onset of cancer, and major risk factors for cardiovascular diseases, such as obesity, increase the prevalence of cancer [49]. The results of a 9.1-year follow-up study in Korea revealed that patients with MetS had a 26% increased stomach cancer risk compared to patients without MetS [50].

Although the results of this study did not analyze specific types of food, regular eating habits, a nutritionally balanced diet, and an increase in vegetables and a decrease in red meat are recommended for a healthy diet related to cancer [51]. Several studies have described changes in eating habits of cancer patients after treatment. In a study conducted on Australian breast CS, meal timing and frequency were improved healthily, and vegetarian-based diets were increasing [52]. Additionally, a meta-study analyzing 14 articles on eating habits in CS found a decrease in red and processed meat. However, consistent results regarding protein, fiber, and supplement intake have not been reported [53]. People who have experienced cancer said they need support and help from family and close people to maintain food cravings and improved eating habits and, furthermore, education from experts and ideas for healthy eating habits are needed [52].

The condition of the patients varied greatly. Patients who were only 1–5 years old, as well as those more than 10 years after cancer treatment, were analyzed together. In addition, because only women were analyzed, there is a limit to this study’s application to specific conditions, such as prostate or testicular cancers, that only exist in men. In future studies, it will be necessary to recruit more patients and closely analyze the incidence of MetS in patients using a longitudinal study that investigates cancer type and duration.

## 5. Conclusions

CS exhibited a higher prevalence of MetS than NC individuals, and the MetS risk increased in CS with high calorie intake, frequent eating out, no diet therapy, and no nutrition education. The prevalence of MetS increased by 1.02–3.07 times in colorectal, cervical, lung, thyroid, and other CS, but there was no significant increase in MetS prevalence in stomach and breast CS. Additionally, low aerobic and muscle strength PA and high sedentary time increased the risk of MetS, whereas high PA decreased the risk of MetS.

## Figures and Tables

**Table 1:** General characteristics of participants.

Variables	NC (*n* = 11,673)		*p*	CS (*n* = 1003)		*p*
	NMetS (*n* = 7351)	MetS (*n* = 4322)		NMetS (*n* = 583)	MetS (*n* = 420)	
NMetS or MetS, %	63.0%	37.0%		58.1	41.9%	
Age, years	57.9 ± 10.9	63.6 ± 9.1	<0.001	58.5 ± 10.6	65.8 ± 9.9	<0.001
Height, cm	156.5 ± 6.2	153.8 ± 5.8	<0.001	156.6 ± 6.0	154.9 ± 5.9	<0.001
Weight, kg	55.7 ± 7.6	60.9 ± 9.0	<0.001	55.4 ± 8.0	61.7 ± 9.2	<0.001
BMI, kg/m^2^	22.7 ± 2.7	25.7 ± 3.3	<0.001	22.5 ± 2.8	25.7 ± 3.2	<0.001
WC, cm	77.4 ± 7.6	87.6 ± 8.2	<0.001	77.4 ± 7.3	87.6 ± 8.2	<0.001
SBP, mmHg	116.2 ± 16.6	130.6 ± 16.7	<0.001	117.2 ± 17.2	128.3 ± 16.1	<0.001
DBP, mmHg	73.7 ± 9.1	75.7 ± 10.2	<0.001	73.5 ± 8.7	76.1 ± 9.7	<0.001
HDL, mg/dL	57.6 ± 12.2	46.6 ± 10.1	<0.001	57.5 ± 12.2	46.4 ± 10.0	<0.001
TG, mg/dL	98.3 ± 50.5	160.2 ± 96.3	<0.001	96.8 ± 43.8	159.9 ± 84.0	<0.001
Glucose, mg/dL	94.3 ± 13.9	113.2 ± 29.8	<0.001	95.2 ± 17.2	111.2 ± 26.3	<0.001
Household income						
High	3771 (51.3%)	2018 (46.7%)	<0.001	312 (53.5%)	187 (44.6%)	<0.001
Medium	1286 (17.5%)	821 (19.0%)		92 (15.8%)	74 (17.5%)	
Low	2294 (31.2%)	1482 (34.3%)		179 (30.7%)	159 (37.9%)	
School						
Middle school	897 (12.2%)	562 (13.0%)	0.410	86 (14.7%)	60 (14.2%)	0.459
High school	3359 (45.7%)	2036 (47.1%)		282 (48.3%)	201 (47.9%)	
College	3095 (42.1%)	1724 (39.9%)		216 (37.0%)	159 (37.9%)	
Smoking						
Current	221 (3.0%)	186 (4.3%)	0.007	20 (3.5%)	13 (3.2%)	0.373
Stopping	257 (3.5%)	190 (4.4%)		41 (7.1%)	36 (8.6%)	
Never	6873 (93.5%)	3946 (91.3%)		521 (89.4%)	370 (88.2%)	
Alcohol						
Low	4359 (59.3%)	2282 (52.8%)	<0.001	374 (64.1%)	247 (58.8%)	<0.001
Medium	1742 (23.7%)	1145 (26.5%)		134 (22.9%)	103 (24.5%)	
High	1250 (17.0%)	895 (20.7%)		76 (13.0%)	70 (16.7%)	
Survivor year						
1–5		-	-	273 (40.9%)	131 (39.1%)	0.054
6–10		-	-	187 (28.0%)	96 (28.7%)	
11–20		-	-	208 (31.1%)	108 (32.2%)	
Cancer number						
1	-	-	-	562 (96.4%)	396 (94.3%)	0.178
2	-	-	-	21 (3.6%)	23 (5.5%)	
3	-	-	-	0 (0.0%)	1 (0.2%)	

Independent *t*-test and chi-square test were used. NC, non-cancer; CS, cancer survivors; NMetS, non-metabolic syndrome; MetS, metabolic syndrome; BMI, body mass index; WC, waist circumference; SBP, systolic blood pressure; DBP, diastolic blood pressure; HDL, high-density lipoprotein cholesterol; TG, triglyceride.

**Table 2:** Odds ratio according to cancer type.

	NC	Total (*n* = 1003)	Stomach (*n* = 111)	Colorectal (*n* = 80)	Breast (*n* = 229)	Cervical (*n* = 190)	Lung (*n* = 17)	Thyroid (*n* = 301)	Other (*n* = 120)
MetS	1.00 (ref)	1.22 (1.07–1.39)	0.98 (0.69–1.45)	1.77 (1.14–2.74)	0.99 (0.75–1.29)	1.72 (1.29–2.30)	3.07 (1.14–5.31)	1.02 (0.89–1.29)	1.45 (1.01–2.07)
WC	1.00 (ref)	1.10 (0.97–1.26)	0.67 (0.43–1.02)	1.64 (1.05–2.55)	0.92 (0.70–1.22)	1.33 (1.01–1.78)	2.14 (0.82–5.54)	1.16 (0.92–1.47)	1.17 (0.81–1.69)
BP	1.00 (ref)	1.09 (0.96–1.24)	1.27 (0.99–2.14)	1.8 (1.14–2.83)	0.83 (0.64–1.08)	1.39 (1.04–1.86)	3.02 (1.44–5.49)	0.91 (0.72–1.14)	1.06 (0.74–1.51)
HDL	1.00 (ref)	1.16 (1.02–1.32)	1.17 (0.80–1.70)	1.34 (0.87–2.09)	0.81 (0.62–1.06)	1.62 (1.21–2.16)	1.13 (0.44–2.94)	1.07 (0.85–1.34)	1.44 (1.01–2.06)
TG	1.00 (ref)	1.14 (1.00–1.30)	0.84 (0.57–1.25)	1.55 (1.00–2.40)	0.99 (0.76–1.30)	1.41 (1.05–1.87)	1.44 (0.56–3.74)	1.05 (0.83–1.32)	1.31 (0.91–1.88)
GLU	1.00 (ref)	1.15 (1.01–1.31)	0.95 (0.65–1.40)	1.08 (0.69–1.69)	1.07 (0.82–1.40)	1.44 (1.08–1.92)	2.98 (1.10–7.06)	1.06 (0.84–1.34)	1.45 (1.01–2.08)

Logistic regression analysis was used. NC, non-cancer; MetS, metabolic syndrome; WC, waist circumference; BP, blood pressure; HDL, high-density lipoprotein cholesterol; TG, triglyceride; GLU, glucose. Forty-five people had two or three cancers.

**Table 3:** Odds ratio according to dietary habit.

Variables	NC		OR (95%CI)	CS		OR (95%CI)
	NMetS (*n* = 7351)	MetS (*n* = 4322)		NMetS (*n* = 583)	MetS (*n* = 420)	
Calorie intake						
Low	3367 (45.8%)	1733 (40.1%)	0.74 (0.68–0.81)	255 (43.7%)	166 (39.6%)	0.77 (0.56–1.05)
Recommended	2323 (31.6%)	1366 (31.6%)	Reference	143 (24.5%)	97 (23.1%)	Reference
High	1661 (22.6%)	1223 (28.3%)	1.43 (1.09–1.79)	185 (31.8%)	157 (37.3%)	1.31 (1.02–1.89)
Three meals per day						
High	4586 (62.4%)	3252 (75.2%)	Reference	430 (69.1%)	314 (74.8%)	Reference
Low	2765 (37.6%)	1070 (24.8%)	1.83 (1.68–1.99)	180 (30.9%)	106 (25.2%)	1.32 (1.03–1.75)
Eating out frequency						
Low	5418 (73.7%)	3177 (73.5%)	Reference	511 (87.7%)	316 (75.2%)	Reference
Medium	1110 (15.1%)	514 (11.9%)	1.01 (0.69–1.07)	39 (6.7%)	51 (12.2%)	1.25 (1.03–1.52)
High	823 (11.2%)	631 (14.6%)	1.11 (1.01–1.64)	33 (5.6%)	53 (12.6%)	2.65 (2.29–3.07)
Breakfast frequency						
High,	5888 (80.1%)	3380 (78.2%)	Reference	513 (88%)	305 (72.6%)	Reference
Medium	750 (10.2%)	445 (10.3%)	1.02 (0.54–1.25)	33 (5.6%)	39 (9.4%)	1.66 (1.36–2.02)
Low	713 (9.7%)	497 (11.5%)	1.17 (0.77–1.76)	37 (6.4%)	76 (18%)	3.37 (2.95–3.86)
Diet supplement						
Yes	4661 (63.4%)	2775 (64.2%)	Reference	461 (79.1%)	342 (81.4%)	Reference
No	2690 (36.6%)	1547 (35.8%)	0.88 (0.72–1.02)	122 (20.9%)	78 (18.6%)	1.09 (0.74–2.35)
Diet therapy						
Yes	2411 (32.8%)	1405 (32.5%)	Reference	183 (31.4%)	113 (26.9%)	Reference
No	4940 (67.2%)	2917 (67.5%)	1.01 (0.77–1.32)	400 (68.6%)	307 (73.1%)	1.24 (1.05–1.85)
Nutrition education						
Yes	515 (7.0%)	229 (5.3%)	Reference	47 (8.1%)	32 (7.5%)	Reference
No	6836 (93.0%)	4093 (94.7%)	1.36 (1.17–1.59)	536 (91.9%)	389 (92.5%)	1.28 (1.08–2.36)

Chi-square test and logistic regression analysis were used. NC, non-cancer; CS, cancer survivors; NMetS, non-metabolic syndrome; MetS, metabolic syndrome; OR, odds ratio; CI, confidence interval.

**Table 4:** Odds ratio according to physical activity.

	NC		OR (95%CI)	CS		OR (95%CI)
Variables	NMetS (*n* = 7351)	MetS (*n* = 4322)		NMetS (*n* = 583)	MetS (*n* = 420)	
Aerobic PA						
High	1775 (24.2%)	921 (21.3%)	0.98 (0.79–1.14)	244 (36.6%)	102 (30.5%)	0.88 (0.67–0.98)
Recommended level	3082 (41.9%)	1616 (37.4%)	Reference	224 (33.5%)	105 (31.3%)	Reference
Low	2494 (33.9%)	1785 (41.3%)	1.37 (1.13–1.71)	200 (29.9%)	128 (38.2%)	1.36 (1.10–1.87)
Strength PA						
High	860 (11.7%)	454 (10.5%)	0.92 (0.74–2.01)	57 (8.6%)	19 (5.7%)	0.78 (0.52–0.96)
Recommended level	515 (7.0%)	294 (6.8%)	Reference	45 (6.7%)	23 (6.8%)	Reference
No	5976 (81.3%)	3574 (82.7%)	1.36 (1.08–1.70)	566 (84.7%)	293 (87.5%)	1.49 (1.07–2.57)
Sedentary time						
Low	3279 (44.6%)	1539 (35.6%)	Reference	268 (40.1%)	139 (41.5%)	Reference
Medium	2744 (37.3%)	1595 (36.9%)	1.24 (1.04–1.45)	256 (38.3%)	108 (32.2%)	1.24 (0.91–1.79)
High	1328 (18.1%)	1188 (27.5%)	1.90 (1.23–3.10)	144 (21.6%)	88 (26.3%)	1.85 (1.39–2.91)

Chi-square test and logistic regression analysis were used. NC, non-cancer; CS, cancer survivors; NMetS, nonmetabolic syndrome; MetS, metabolic syndrome; OR, odds ratio; PA, physical activity; CI, confidence interval.

## Data Availability

This study analyzed data released from government agencies: [https://knhanes.kdca.go.kr] (accessed on 15 April 2023).

## Abbreviations

PA	physical activity
CS	cancer survivors
MetS	metabolic syndrome
NC	non-cancer
CI	confidence interval
BP	blood pressure
WHO	World Health Organization
